# Preparation and Evaluation of Eudragit® L100 Nanoparticles Loaded Impregnated with KT Tromethamine Loaded PVA -HEC Insertions for Ophthalmic Drug Delivery

**DOI:** 10.15171/apb.2019.068

**Published:** 2019-10-24

**Authors:** Ghobad Mohammadi, Shahla Mirzaeei, Shiva Taghe, Pardis Mohammadi

**Affiliations:** Pharmaceutical Sciences Research Center, Health Institute, Kermanshah University of Medical Sciences, Kermanshah, Iran.

**Keywords:** Ketorolac tromethamine, Inserts of Eudragit ®L100 nanoparticles, Ophthalmic drug delivery, PVA

## Abstract

***Purpose:*** The purpose of the present study was to improve the ocular delivery for ketorolac tromethamine (KT) used to treat inflammation of the eye.

***Methods:*** Eudragit nanoparticles loaded with KT were prepared and incorporated in polyvinyl alcohol (PVA) and hydroxyethyl cellulose (HEC) films. Nanoparticles were characterized by Fourier transform-infrared (FT-IR), scanning electron microscopy (SEM). Physicochemical properties and encapsulation effciency were investigated for nanoparticles. Also, the inserts were evaluated for their physiochemical parameters like percentage moisture absorption, percentage moisture loss, thickness and folding endurance.

***Results:*** Mean particle size and zeta potential were in range of 153.8-217 nm and (-10.8) - (-40.7) mV, respectively. The results show that the use of a surfactant has not led to any major change on drug loading. The loading increases with the amount of polymer. The insert had a thickness varying from 0.072 ± 0.0098 to 0.0865 ± 0.0035 mm. The thicknesses of the inserts and the folding endurance increased with the total polymer concentration. The physicochemical properties showed that the Eudragit® L-100 nanoparticles loaded PVA-HEC films could be an effective carrier for KT.

***Conclusion:*** For the first time, inserts of Eudragit nanoparticles were successfully prepared for ophthalmic drug delivery system to prevent frequent drug administration and enhance patient compliance.

## Introduction

Ocular inflammation can be caused by a wide variety of factors including autoimmune disease, infection, allergies, injury or trauma, and surgery. Normally, the treatment of ocular inflammation involves the use of corticosteroids and non-steroidal anti-inflammatory drugs (NSAIDs). Although corticoids have more or less lipophilic characters, NSAIDs are weak acids that are ionized at the lachrymal fluid pH level, a fact that limits their corneal permeability.^1,2^ Ketorolac tromethamine (KT) is a non-steroidal anti-inflammatory drug from the family of heterocyclic acetic acid derivatives.

Drug delivery systems decrease the side effects of drugs such as postoperative inflammation of the eyes, postoperative pain and the conjunctivitis with no alteration of corneal opacity.^3,4^ Despite eye drops cause a little blurring, they are popular due to low cost, great simplicity of formulation, development and production and better acceptance by patients. According to the physiology and anatomy of the eye, the prescription drug was absorbed in a very small percentage due to the protection mechanisms, such as tearing and blinking reflex.^5-7^ Polymer nanoparticles are reported to be devoid of any irritant effect on cornea, iris, and conjunctiva and thus appear to be a suitable inert carrier for ophthalmic drug delivery.

A Eudragit nanoparticle suspension is one of the most significant carrier systems for the ophthalmic release of non-steroidal anti-inflammatory drugs such as ibuprofen, flurbiprofen and prednisolone.^8-10^ Ophthalmic inserts are solid devices intended to be placed in the cul-de-sac or on the cornea which represent one of the possibilities to reach increased residence time. Also, they present the advantage of avoiding a pulsed release due to multiple applications. Design, construction and technology of ocular insert in a controlled and sustained ocular delivery device are gaining rapid improvement to overcome this constraint.^11-14^

Inserts have been used for non-steroidal anti-inflammatory ophthalmic drug delivery in several reports. KT inserts were prepared using different polymers such as hydroxy propyl methylcellulose, ethyl cellulose, methylcellulose and polyvinyl pyrrolidone in different proportions, to improve residence time and corneal absorption.^15-17^

In this study, Eudragit^®^ L100 nanoparticles loaded with KT were prepared and characterized. The insert is, in effect, a device used to prolong the contact time of the nanoparticles with the corneal insert. The objective of the study which is the incorporation of Eudragit^®^ L100 nanoparticles in hydroxyethyl cellulose (HEC) and polyvinyl alcohol based on the insert has been investigated for ophthalmic drug delivery system.

## Materials and Methods

### Materials

HEC and polyvinyl alcohol (PVA) were purchased from Sigma–Aldrich Chemical. Eudragit^®^ L100 polymer was obtained from Rofarma Italia S.r.l. (Rofarma, Gaggiano, Italy). KT was a kind gift from Sina-Daru (Sina-Daru, Iran). All the other reactants were of analytical grade or higher.

### Preparation of the nanoparticles

The nanoparticles were prepared adapting the spontaneous emulsiﬁcation technique previously described by Bodmeier et al.^18^ Specified amounts of Eudragit^®^ L100 polymers (0.5 and 1 mg/mL) and drugs (0.1 mg/mL) were dissolved in 4 mL of acetone or, dimethyl sulfoxide (DMSO) at room temperature. This organic phase was slowly poured into 10 mL of aqueous phase (distilled water) containing 1% (w/v) PVA or 1% (w/v) PVA and 0.2% (w/v) Tween 80 (as a hydrophilic emulsifier) under moderate magnetic stirring at 800 rpm and the nanoparticles were spontaneously formed. The suspension was stored in a screw-top amber glass container in the refrigerator (+4°C) until use. The process conditions and formulation factors were investigated, in order to obtain the nanoparticles with suitable size and zeta potential. The compositions of the experimental formulations are shown in Table 1.

**Table 1:** Preparative characteristics of the different formulations of KT

**Formulation**	**PVA (%)**	**Tween 80 (%)**	**Eudragit (%)**	**Drug (mg/mL)**	**Solution**	**HEC (mg/mL)**
K1	1	0.20	1	0.1	Acetone	0.1
K2	1	-	1	0.1	Acetone	0.1
K3	1	-	1	0.1	DMSO	0.1
K4	1	-	0.5	0.1	Acetone	0.1

### Preparation of solutions for ocular inserts

**Figure 1:**
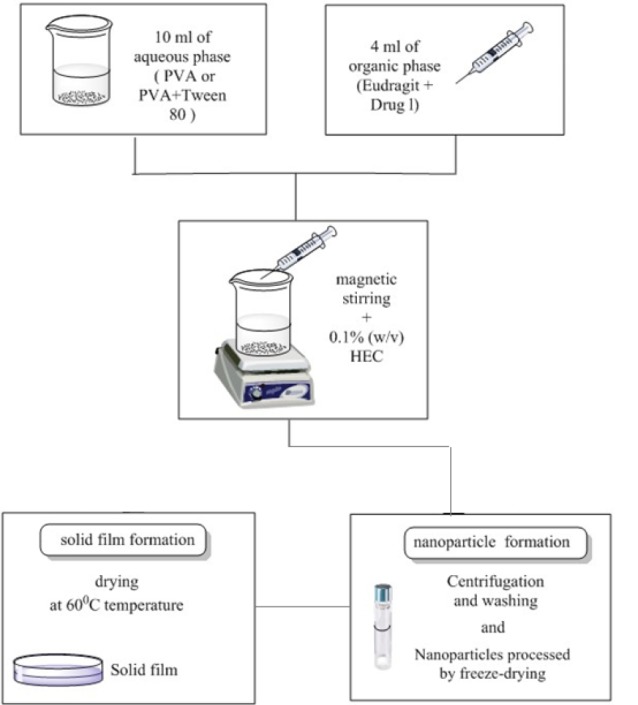
The experimental procedure for nanoparticle and solid insert.

The nanoparticles incorporated in the structure of the film were prepared by addition of 0.1% (w/v) HEC in 14 ml of nanoparticle solution (recently synthesized) under magnetic stirring for 1 hour. The solutions were poured in a mold covered for film casting preparation and were placed on a leveled surface at 60°C temperature and let dry for 24 hours. The experimental flow sheet is shown in Figure 1. After drying, the films were removed and conditioned in sealed plastic bags stored at room temperature.^19^

### Evaluation of nanoparticles

#### Particle size and zeta potential measurements

Particle size and zeta potential were determined by photon correlation spectroscopy and laser-doppler anemometry using a Malvern Zeta sizer 3000 HS (Malvern Instruments Ltd., Malvern, UK). Nanoparticle suspensions were analyzed at 25°C. All analysis was performed in triplicate and the average value was considered for data analysis of zeta potential.

#### Determination of drug loading and entrapment efficiency of KT

KT was measured loading percentage of nanoparticles as follows: 10 mg of lyophilized KT nanoparticles were mixed with 5 mL of distilled water and then were dissolved in 5 mL of methanol. The encapsulation efficiency of the nanoparticles was determined according to Motwani et al.^20^ Drug-loaded nanoparticles were separated from the aqueous medium containing non-associated KT using Beckman Coulter Optima L-90K Ultracentrifuge (Beckman Coulter, USA) at 30 000 rpm and 4°C for 30 minutes. KT concentration in the supernatant was measured by UV spectrophotometry at 323 nm. The drug loaded into the nanoparticles was calculated as the difference between the total amount of drug used for the preparation of the nanoparticles and the amount of drug found in the supernatant. Each measurement was repeated for three times.^21^ Drug loading and entrapment efficiency *was* calculated *using* following equations (Eqs. 1 and 2):

(1) $$\%DL=\frac{Total\;amount\;of\;Ketorolac-\;Free\;drug}{Weight\;of\;nanoparticl}\times 100\;\left(1\right)\;$$

(2) $$\%EE=\frac{Total\;amount\;of\;Ketorolac-free\;drug\;}{Total\;amount\;of\;Ketorolac}\times 100\;\left(2\right)\;$$

### Evaluation of insert

#### Thickness measurement

The thickness of the inserts (n= 3) was measured using screw a screw thickness gauge (0.01 mm least count) at different spots of the patches.^22^

### Moisture uptake

The percentage moisture uptake test was carried out to check physical stability or integrity of inserts. Inserts were weighed accurately and placed in a desiccator containing 100 mL of saturated solution of aluminum chloride (79.5% RH). After 3 days, the inserts were taken out and reweighed. The percentage of moisture uptake was calculated as the difference between the final and initial weight with respect to the initial weight (Eq. 3).^23^

(3) $$\%moisture\;uptake=\frac{Final\;weight-Initial\;weight}{Initial\;weight}\;$$

The percentage moisture loss was carried out to check integrity of the insert at dry condition. Inserts were weighed and kept in a desiccator containing anhydrous calcium chloride. After 3 days, the inserts were taken out and reweighed, the percentage moisture loss was calculated using the formula (Eq. 4).^23^

(4) $$\%moisture\;loss=\frac{Initial\;weight-\;Final\;weight}{Initial\;weight}$$

### Folding endurance

The folding endurance was expressed as the number of folds (number of times the insert could be folded at the place without breaking gave the exact value and evaluated the ability of the sample to with stand folding. A strip of inserts (2 cm^2^) was cut evenly and repeatedly folded at the same place till it broken.^24^

### Swelling percentage

Swelling of the polymer depends on the concentration of the polymer, ionic strength and the presence of water. To determine the swelling index of prepared ocular inserts, initial weight of insert was taken, and then placed in agar gel plate (2% m/v agar, pH 7.2) and incubated at 37 ± 1°C. Insert was removed from plate after one hour, surface water was removed with help of filter paper, and insert was reweighed. Swelling index was calculated (Eq. 5).

(5) $$\%Swelling\;Percentage=\frac{Wt-W0}{W0}\times 100$$

W_t_: weight of swollen insert after time t

W_0_: original weight of insert at zero time

### SEM studies

Scanning electron microscopy (SEM) was used to observe the morphology of the nanoparticles and inserts. The fracture surfaces were analyzed using a high-resolution, field emission scanning electron microscope (HR-FE-SEM) MIRA 3 XMU from TESCAN Company. The SEM is equipped with the unique real-time ‘‘In-Flight Beam Tracing^™^’’ technique for the performance and spot optimization. Furthermore, the SEM provides a unique live stereoscopic imaging using an advanced 3D beam technology. The samples were sputter-coated with a gold layer and kept clean, dry and placed on metal stubs with adhesive tape and then observed under a scanning electron microscope.

### Fourier transform-infrared analysis

Fourier transform-infrared (FT-IR) spectra were obtained using FTIR-spectrometer (Shimadzu, Japan). Samples were dried in a vacuum desiccator, mixed with micronized KBr powder and compressed into discs using a manual tablet press. FTIR spectra of pristine KT, Eudragit^®^ L100, HEC, PVA, Teen 80, Eudragit^®^ L100 nanoparticles and inserts with Eudragit^®^ L100 nanoparticles were obtained.

### In vitro cytotoxicity test

The tetrazolium-based colorimetric assay (MTT test) was employed to evaluate the in vitro cytotoxicity of nanoparticles against L929 (mouse fibroblast) cells (ATCC). Briefly, cells were seeded in 96-well plates at a density of 1×10^4^ cells/well in 0.1 mL growth medium. Then, 0.1 mL of nanoparticles (0-100 µg/mL) were added to wells and co-incubated for 24 hours. After 24 hours, 20 µL of MTT solution (5 mg/mL) was added to each well and incubated for another 2 hours. The MTT solution was then carefully removed from each well, and 150 µL DMSO was added to dissolve the MTT formazan crystals. The absorbance was recorded at 570 nm using an ELISA microplate reader (Bio-Rad, Hercules, CA, USA). Cell viability (%) was calculated according to the following equation:

Cell viability (%) = absorption test/absorption control × 100,

The absorption control was absorbance from control wells. All data are expressed as the mean of six measurements (mean ± SD, n = 6).

### Drug release study

The in vitro drug release of the inserts was determined by dialysis method in sink conditions.

Sink condition is the ability of the dissolution media to dissolve at least 5 times the amount of drug and water solubility of ketorolac is 0.513 mg/mL.

The prepared samples were placed in the dialysis bag (Mw cut-off = 12 000–14 000 Da; Delchimica Scientific Glassware, Milan, Italy) containing 1 mL of phosphate buffer at pH 7.4. The dialysis bag was then kept in release medium 40 mL of the same aqueous buffer and incubated at 37°C with continuous orbital mixing (100 rpm). Phosphate buffer solution (Dissolve 13.6 g of KH2PO4 and 0.4 g of NaOH in distilled water and dilute to 1 L, pH 7.4) was prepared according to the US Pharmacopeia. Samples were collected periodically from the shaker and replaced with fresh dissolution medium and the drug concentration was quantified in the acceptor phase. The samples were collected into filters and KT on centration was determined spectrophotometrically at λ_max_ of 323 nm. These studies were performed in triplicate for each sample, but average values were considered in data analysis and graphical presentations.

The kinetics of drug release was fitted into four types of mathematical models including zero order, first order, Higuchi square root, and Peppas to find out the mechanism of drug release. The kinetic model which displayed maximum squared correlation coefficient (RSQ) and minimum prediction error (PE) was selected as the best kinetic model.

### Statistical analysis

All the experiments were done in triplicate. Results are expressed as mean ± SD. The statistical analyses were performed using the SPSS software package (SPSS Inc., 1999). Kruskal-Wallis and *t* tests were used to determine statistical significance of results. Differences were considered significant for values of *P* < 0.05.

## Results and Discussion

### Encapsulation efficiency and drug loading

The drug loading and the entrapment efficiency percentages of nanoparticles are reported in Table 2. It is evident from Table 2 that the entrapment efficiency percentages and drug loadings were not significantly different between K1, K2 and K4 but decreased in K3 formulation. Also, the results show that the use of Tween 20 as a surfactant has not led to any major changes on entrapment efficiency and drug loading. The entrapment efficiency was affected by the amount and molecular weight of Eudragit^®^ L100 in the blend.^25^ Improvement in the entrapment efficiency percentage and drug loading in formulation K4 may be caused by the use of acetone as a solvent and the decrease in the relative amount of Eudragit^®^ L100, respectively. The entrapment efficiency percentages and drug loading were boosted by decreasing the ratio of drug: polymers.

**Table 2:** Physicochemical properties of ocular nanoparticles of KT (mean ± SD, n = 3)

** Formulation**	** Size (nm) **	**polydispersity index **	**Zeta potential (mV) **	**Entrapment efficiency(%)**	** Drug Loading (%)**
Nanoparticles of K1	217.5 ± 0.11	0.226 ± 0.013	-40.7 ± 3.1	40.99	32.79
Nanoparticles of K2	215.75 ± 0.12	0.215 ± 0.049	-16.2 ± 4.8	36.3	29.1
Nanoparticles of K3	216.3 ± 0.24	0.248 ± 0.031	-10.8 ± 2.9	20.66	16.5
Nanoparticles of K4	153.7 ± 0.01	0.318 ± 0023	-16.9 ± 1.5	45.4	36.3

### Evaluation of nanoparticles: size, morphology and zeta potential

To produce nanoparticles with the desired properties such as small particle size, a low polydispersity index (PDI), and high loading efficiency, different formulations and process parameters were examined. Size, PDI and zeta potential nanoparticles are reported in Table 2. Mean particle size and zeta potential were in the range of 153.8-217 nm and (-10.8) - (- 40.7) mV, respectively. The results showed that there was no noteworthy difference between the size and PDI of K1, K2, and K3. The mean particle size of K4 was found decrease because of Eudragit^®^ L100 amount.

**Figure 2:**
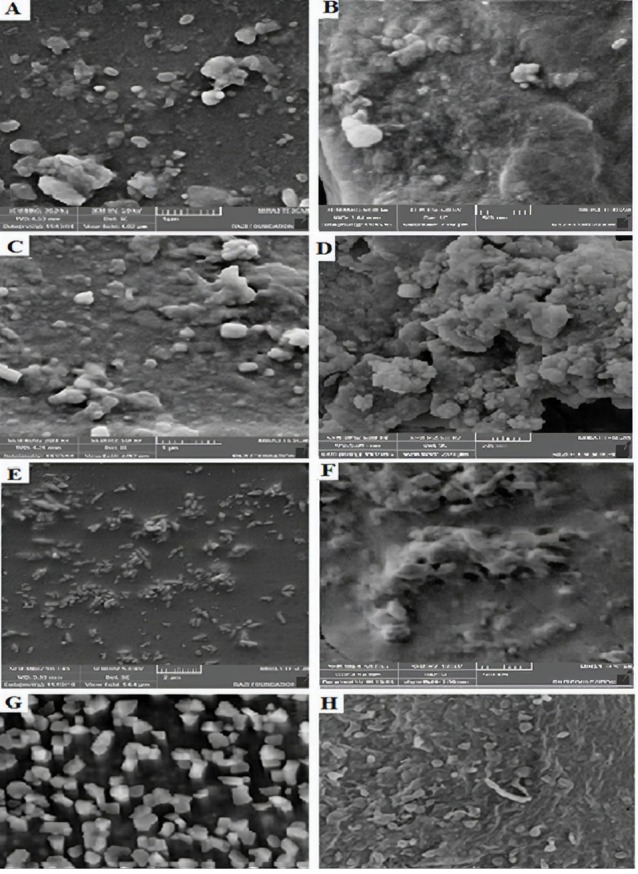
SEM images of K1 formulation insert (A), K1 formulation nanoparticles(B), K2 formulation insert (C), K2 formulation nanoparticles (D), K3 formulation insert (E), K3 formulation nanoparticles (F), K4 formulation insert (G), and K1 formulation nanoparticles (H).

The presence of Tween 20 in this formulation may cause high negative zeta potential values of K1. High zeta potentials lead to an increase in aggregate stability. High negative zeta potential values are expected for pure anionic polymer (Eudragit^®^ L100, etc) nanoparticles due to the presence of carboxyl groups on the polymeric chain extremities.^25^ The SEM photograph verified that particles were in the nano-range with no visible aggregation (Figure 2). The mean particle size of Eudragit^®^ L100 nanoparticles was found to increase by Eudragit^®^ L100 concentration. Enhancement in the concentration of the dissolved Eudragit^®^ L100 polymer increased the viscosity of organic phase and reduced the stirring efficiency resulted in the formation of the bigger emulsion drople.^26^

The factor which might be responsible for such an effect can be the presence of residual PVA on the nanoparticles surface.^27^ Also, a higher viscosity of the organic phase causes a better distribution of the drug in the matrix. On the contrary, lowering the viscosity of the organic phase allows drugs to come close to the surface during particles formation and dissolve in the surrounding aqueous medium, resulting in lower drug content.^6^

### Evaluation of ocular insert

The prepared KT ocular inserts were evaluated or characterized based upon their physicochemical characteristics such as moisture loss, moisture uptake, thickness, and swelling percentage folding endurance. These results were shown in Table 3. The insert had a thickness varying from 0.072 ± 0.0098 to 0.0865 ± 0.0035mm. It was found that the thicknesses of the inserts grew with increase in the total polymer concentration.

**Table 3:** Physicochemical parameters of the ocular inserts of KT (mean ± SD, n = 3)

**Formulation**	**Thickness (mm)**	**Folding endurance**	**Moisture loss (%)**	**Moisture uptake (%)**	**Swellingpercentage(%)**
K1	0.073 ± 0.0070	163 ± 5	4.095 ± 0.13	3.858 ± 0.07	123.09 ± 0.974
K2	0.0865 ± 0.0035	138 ± 2	3.797 ± 0.06	2.816 ± 0.14	116.03 ± 1.33
K3	0.0845 ± 0.0007	253 ± 6	5.494 ± 0.09	1.055 ± 0.13	28.16 ± 1.24
K4	0.070 ± 0.0098	104 ± 3	6.122 ± 0.11	5.294 ± 0.06	111.5 ± 0.847

Among all the formulations, the high value of moisture uptake can be observed in K4.

Ophthalmic insert results showed that folding endurance was in the range of 138 ± 2 to 253 ± 6. The folding endurance was found to be lowest for formulation K4 (104 ± 3). It was found that the folding endurance decreased the percentage of Eudragit^®^ L100 declined. The folding endurance was discovered to be the highest for formulation K3 (253 ± 6), which was due to the presence of residual DMSO. The equilibrium swelling percentage varied from 28.16 ± 1.24 (formulation K3) to 123.09 ± 0.974 (formulation K1). The low value of swelling percentage can be observed in K3 due to the use of DMSO behavior as the solvent.

High value of moisture uptake for all formulations is caused by the decreasing swelling behavior of Eudragit^®^ L100. Low value of moisture uptake can be observed in K3 due to the use of DMSO behavior as the solvent.

### FT-IR analysis

**Figure 3:**
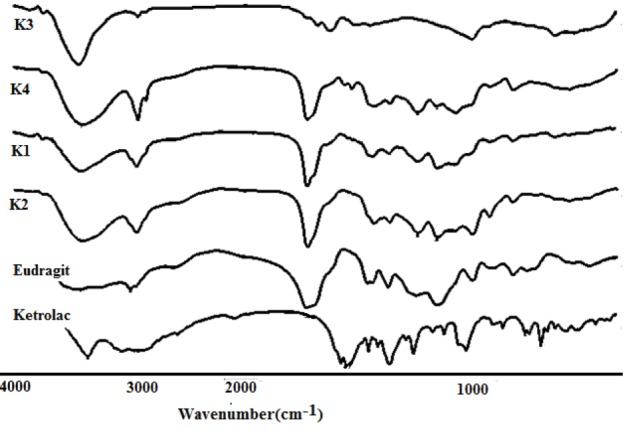
FT-IR spectrum: KT; Eudragit; K1; K2; K3 and K4 nanoparticles.

**Figure 4:**
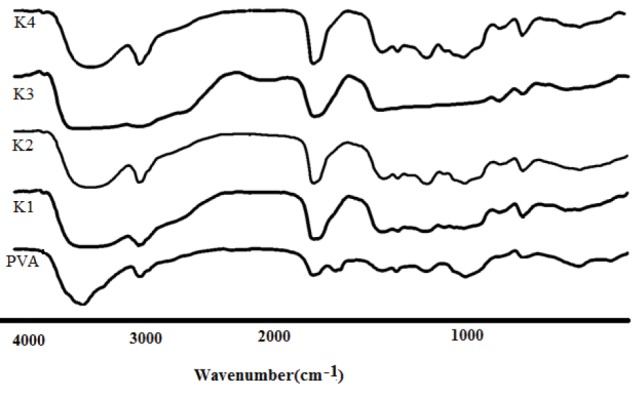
FT-IR spectrum: PVA; K1; K2; K3 and K4 inserts.

The FT-IR results are shown in Figures 3 and 4. The FT-IR spectrum of KT showed a peak at 3352.28 cm^-1^ assigned to N-H and NH stretching vibration. Peaks at 1469.76 cm^-1^ and 1431.1 cm^-1^ corresponded to C =C aromatic and aliphatic stretching vibrations. Stretching vibration observed at 1381.03 cm^-1^ is a characteristic of -C-N stretching vibration. Peak at 1049.28 cm^-1^ is due to -OH bending confirms presence of alcoholic group.

The IR of Eudragit^®^ L100 displayed several characteristic bands at 1735 cm^−1^ (esteriﬁed carboxyl groups vibrations), 1180 cm^−1^ and 1265 cm^-1^ (ester vibrations), 1385cm^−1^, 1473.62 cm^-1^ and 2989.66 cm^-1^(CH_X_ vibrations), and 3240 cm^−1^ (OH groups vibrations). In the nanoparticles spectrum, ester vibrations group of Eudragit^®^ L100 was observed at 1265 and 1180 cm^-1^. The intensity of hydroxyl group absorption bands around 3400 cm^−1^ is enhanced owing to the synergistic effect of KT and Eudragit^®^ L100. The vibrational peak appearing at 848.68 cm^-1^ was assigned to C–H rocking mode of PVA, which appears in the peak of inserts. ACH & CH asymmetric stretching vibration of PVA appearing at 2924 cm^-1^ is shifted to 2935 cm^-1^ in the inserts.^28^ From the FT-IR results, the overlapping between amide groups of the KT and carbonyl group of Eudragit^®^ L100 were appeared in the spectrum of nanoparticles. Peak at 1049.28 cm-1is due to -OH bending confirm presence of alcoholic group, and peaks at 705.95 cm^-1^, 729.09 cm^-1^, 798.53 cm^-1^ confirms C-H bending (Aromatic) thus confirms the structure of KT tromethamine.^29^

### In vitro cytotoxicity test

**Figure 5:**
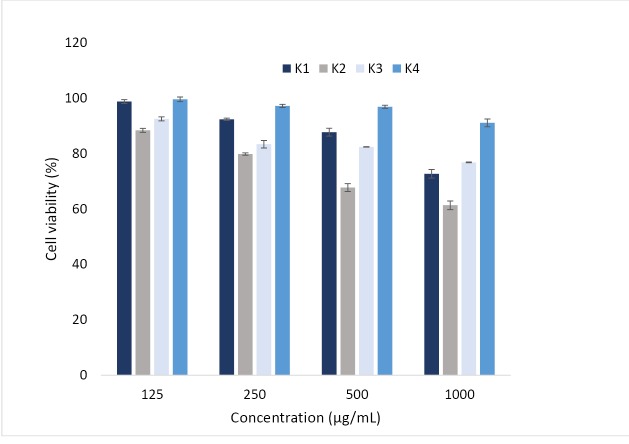
Cell viability of cells after treatment with different concentrations of Eudragit NPs. Cell viability measured by MTT assay. Data are expressed as the mean ± SEM of three separate experiments (n = 6).

Preliminary studies showed that the developed ocular inserts with nanoparticles were non-irritating to the rabbit eyes after administration of ocular inserts (Figure 5). However, whether the prepared inserts were safe for ocular drug delivery should be carefully evaluated before further applications. The cytotoxicity of ocular inserts was investigated in L929 (mouse fibroblast) cells (ATCC). After 24 hours, cell viability was greatly dependent on the concentration of nanoparticles, especially in K1, K2 formulations. The cell viability decreased drastically for all of formulation with increasing the concentration of nanoparticles. As a result, the developed nanoparticles with low cell cytotoxicity could be regarded as safe drug carriers for ocular drug delivery. Similar results were also reported by, whom demonstrating L929 (mouse fibroblast) cells were an ocular carrier for cyclosporine A loaded nanoparticles.^30^

### In vitro KT release study

**Figure 6:**
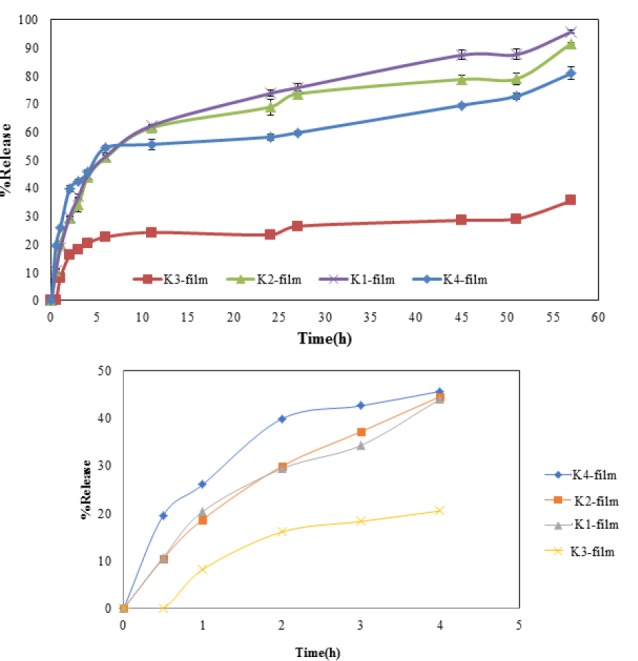
In vitro release profile of KT from K1; K2; K3 and K4 inserts. Release assay was performed in phosphate-buffered saline (PBS) solution, pH: 7.4, at 37°C with agitation (n = 3).

KT release profiles from the formulated KT-polymer nanoparticles are shown in Figure 6. The KT percentage of release within the first 3 hours for K4 formulation and 4 hours for K1, and K2 formulations were almost 40%. Also, the KT percentage of release within the first 3 hours for K3 was 18%. In the initial burst release, KT release from *hydrophilic* polymeric *matrix*. The second stage lasted 50 hours; the release rate was declined for nanoparticles due to the fact that the drug releases by dissolution and diffusion. The high molecular weight of Eudragit^®^ L100 increases the sedimentation viscosity. Therefore, the release rate increases with *an increase* in the amount of Eudragit^®^ L100. The reason for the high initial release rate of the nanoparticles might be due to the distributed drug being near the surface of inserts.^31^ The release patterns showed that the drug release depends on the type of solution and the amount of polymers in the blend. Decreasing the amount of the Eudragit^®^ L100 led to a decrease in the drug release rate. This result is in agreement with the results reported by Adibkia et al.^32^ Also, Figure 6 shows a decrease in release rate when DMSO is used as solution in formulation K3. This is may be due to the presence of intermolecular interactions.

To clarify the mechanism of release, the release data of microfiber formulations were fitted into various kinetic models. The prediction ability of the kinetic models was compared by calculation of RSQ and PE. Considering the RSQ and PE values, Higuchi model was the best fitting model for K1, K2, K3 formulations and Peppas was the best kinetic model for formulation K4.

## Conclusion

For the first time, KT loaded Eudragit^®^ L100 were successfully prepared by spontaneous emulsiﬁcation technique. The obtained results showed that Eudragit^®^ L100 could be a useful nanocarrier for non-steroidal anti-inflammatory drugs such as KT. For the first time, the of Eudragit^®^ L100 nanoparticles in PVA and HEC matrix was used as insert films. The results indicated that there was no noteworthy difference between the sizes of the nanoparticles. The increase in size of nanoparticles could be due to increasing the amount of Eudragit^®^ L100. Formulation K1 showed higher zeta potentials (-40.7 ± 3.1) than other formulations because of the presence of Tween 20. The entrapment efficiency percentages and drug loading were declined with decreasing the ratio of drug: polymers. The results showed that the addition of surfactant could not change the loading and entrapment efficiency. Moreover, improving the entrapment efficiency percentage and drug loading in formulation K4 may be due to the use of acetone as a solvent. Finally, formulation K4 was selected the best formulation for therapeutic study. As a result, it can be stated that this form of insert is suitable for ophthalmic drug delivery because of flexibility, smooth and softness and high release strength.

## Ethical Issues

Not applicable.

## Conflict of Interest

There is no conflict of interest.

## Acknowledgments

The authors would like to acknowledge the Research Council of Kermanshah University of Medical Sciences (Grant number: 95522) for financial support of this work.
